# The Case for S2: The Potential Benefits of the S2 Subunit of the SARS-CoV-2 Spike Protein as an Immunogen in Fighting the COVID-19 Pandemic

**DOI:** 10.3389/fimmu.2021.637651

**Published:** 2021-03-09

**Authors:** Priyanka Shah, Gabriela A. Canziani, Erik P. Carter, Irwin Chaiken

**Affiliations:** Department of Biochemistry and Molecular Biology, Drexel University College of Medicine, Philadelphia, PA, United States

**Keywords:** SARS-CoV-2, S2 subunit, COVID-19, coronavirus, spike protein, antibodies, immunity, SARS-CoV-2 vaccine

## Abstract

As COVID-19 cases continue to rise, it is imperative to learn more about antibodies and T-cells produced against the causative virus, SARS-CoV-2, in order to guide the rapid development of therapies and vaccines. While much of the current antibody and vaccine research focuses on the receptor-binding domain of S1, a less-recognized opportunity is to harness the potential benefits of the more conserved S2 subunit. Similarities between the spike proteins of both SARS-CoV-2 and HIV-1 warrant exploring S2. Possible benefits of employing S2 in therapies and vaccines include the structural conservation of S2, extant cross-reactive neutralizing antibodies in populations (due to prior exposure to common cold coronaviruses), the steric neutralization potential of antibodies against S2, and the stronger memory B-cell and T-cell responses. More research is necessary on the effect of glycans on the accessibility and stability of S2, SARS-CoV-2 mutants that may affect infectivity, the neutralization potential of antibodies produced by memory B-cells, cross-reactive T-cell responses, antibody-dependent enhancement, and antigen competition. This perspective aims to highlight the evidence for the potential advantages of using S2 as a target of therapy or vaccine design.

## Introduction

The COVID-19 pandemic continues to be a global public health threat. As of January 31, 2021, there have been over 102 million confirmed cases and over 2.2 million confirmed deaths worldwide (1). It is imperative to learn more about antibodies and T-cells produced in response to the severe acute respiratory syndrome coronavirus-2 (SARS-CoV-2) in order to develop effective therapies and ultimately a vaccine (2). Current antibody and vaccine research focuses heavily on the receptor-binding domain (RBD) of the S1 subunit of the SARS-CoV-2 spike protein (S) and the S1 more broadly. However, based on antibody neutralization studies of a structurally-similar protein, the envelope protein (Env) of human immunodeficiency virus-1 (HIV-1), it is possible that potent neutralizing antibodies (nAb) against S2, which is somewhat analogous to HIV's gp41, exist and can be utilized for therapeutics and vaccine development.

SARS-CoV-2, a betacoronavirus, is a member of the Coronaviridae family, which consists of enveloped, positive-sense single-stranded RNA viruses (3–5). While all of the human coronaviruses can be pathogenic, variation in symptom severity is broad. Human coronaviruses (HCoV) OC43, HKU1, NL63, and 229E, known as the “common cold coronaviruses,” cause mild symptoms, while Middle East respiratory syndrome coronavirus (MERS-CoV) and severe acute respiratory syndrome coronavirus (SARS-CoV) can cause severe and even fatal symptoms, including viral pneumonia. COVID-19, the disease caused by SARS-CoV-2, often leads to cough, fever, and fatigue, among other symptoms (3, 5). However, severity of disease can range from completely asymptomatic to fatal. A literature review of 21 studies found that of individuals who tested positive for COVID-19, the percentage of asymptomatic individuals ranged from 5 to 80% (6). This varying percentage poses difficulties in reducing transmission (3, 5). Additionally, the spike protein of SARS-CoV-2, S, has a 10 to 20 times greater affinity for ACE2 than that of SARS-CoV, which may contribute to greater infectivity (5).

SARS-CoV-2 can spread via respiratory droplets, inhaled aerosols, or ocular contact (5, 7). Fecal-oral transmission is also possible (5). Infection leads to increased serum levels of IL-4, IL-10, IL-1β, IFN-γ, MCP-1, and IP-10 and can progress to acute respiratory distress syndrome and a cytokine storm, promoting inflammation and acute lung injury (4, 8).

**Figure 1:**
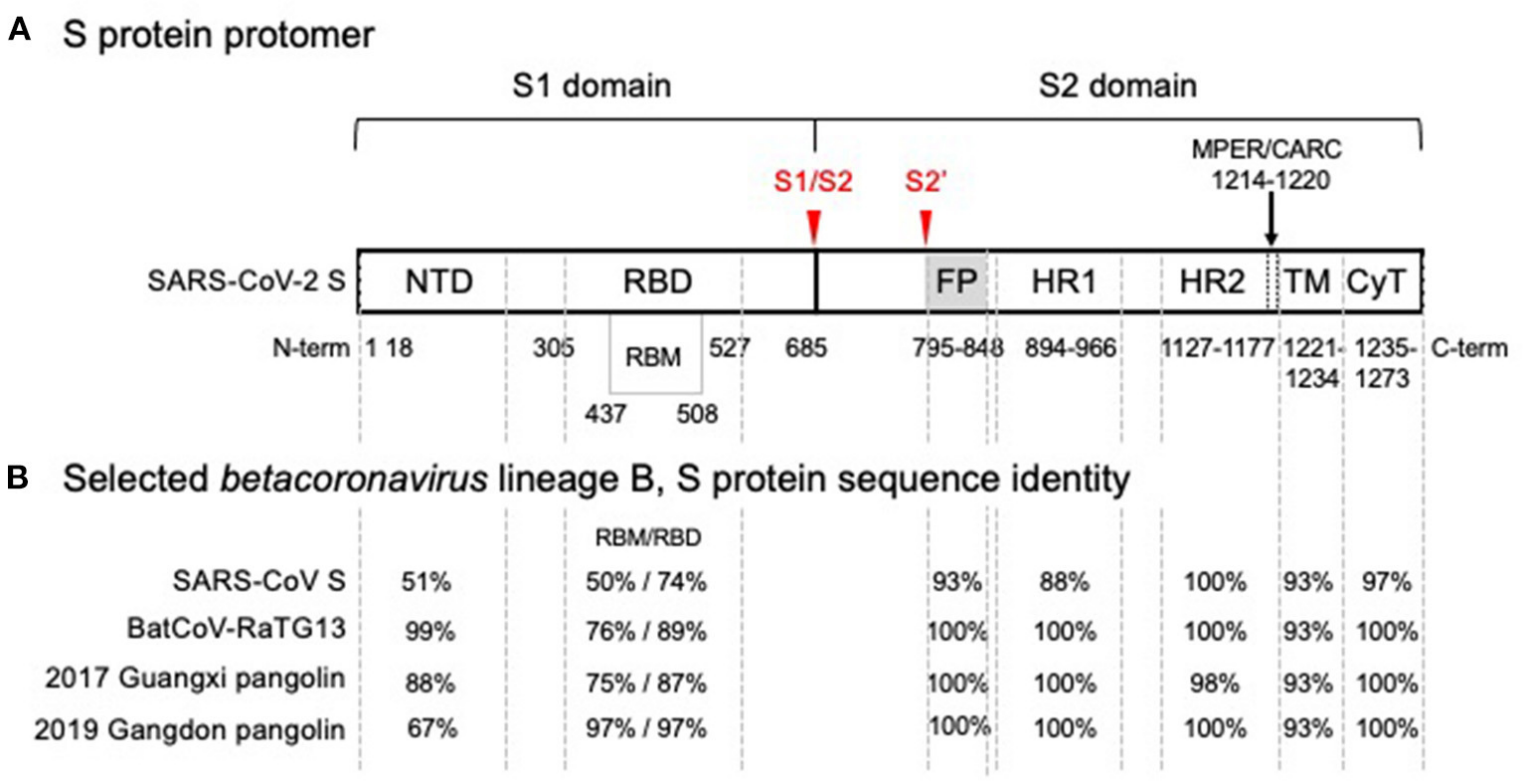
SARS-CoV-2 spike glycoprotein monomer representation showing **(A)** functional domains and **(B)** comparison of amino acid sequence identity with SARS-CoV and related isolates in the wild. **(A)** Functional domain S1 mediates binding of the receptor binding domain (RBD) to the angiotensin converting enzyme 2 (ACE2), the host cell receptor that is specifically recognized by the receptor binding motif (RBM) interface, is cleaved (S1/S2) and shed. Shedding exposes the S2 domain. Cleavage at S2' triggers spike trimer activation, release of the fusion peptide (FP), heptad repeat 1 (HR1) and heptad repeat 2 (HR2); the membrane proximal external region (MPER) is sometimes considered part of HR2 and with a cholesterol recognition/interaction amino acid consensus (CARC) sequence, potentially participating in membrane lipid fusion. The transmembrane domain (TM) and a short cytoplasmic tail (CyT) are indicated. Cleavage sites that drive host-cell infection are shown in red. **(B)** Phylogenetic analysis of SARS-CoV-2 domain sequences identity among SARS-CoV-2, bat and pangolin's isolates, and SARS-CoV are tabulated for each domain. A high degree of sequence identity for the RBD in SARS-CoV correlates well with ACE2 receptor recognition. However, the RBM-ACE2 interface is 50% identical and may account for the increased ACE2 binding affinity. Sequence identities are high for all functional segments of S2, the Type I metastable domain that induces viral-host cell membrane fusion, suggesting an optimum sequence-structure-function relationship. Metastability is a functional requirement, allowing these proteins to refold into a lower energy conformation while transferring the difference in energy to catalyze the membrane fusion reaction. Structural studies have shown that stable immunogens presenting the same antigenic sites as the labile wild-type proteins efficiently elicit potent neutralizing antibodies. In the alternative endosomal pathway that is apparently mediated by NTD, the fusion machine of S2 is equally required to infect the host cells (9, 10). Furthermore, detrimental amino acid substitutions found in the S2 domain of Hepatitis Mouse Virus, but not in SARS-CoV-2, affected the post fusion conformational stability, explaining a reported reduction of S-mediated membrane fusion (11).

The genome of SARS-CoV-2 encodes 4 structural proteins, namely the nucleoprotein (N), the membrane glycoprotein (M), the small envelope glycoprotein (E), and the spike protein (S), in addition to 16 non-structural proteins (4). S, which is used to enter cells, is a trimer with protomers, each composed of two subunits, S1 and S2 (see Figure 1). S1 contains an exposed receptor-binding domain (RBD) that binds ACE2 receptors while S2, which is not fully exposed until after receptor binding, is necessary for fusion of viral and host membranes (3). The RBD is a less conserved region of S, while S2 is markedly more conserved across coronaviruses (12). S2's greater structural conservation could prove beneficial for therapeutic and vaccine design (13).

To develop a successful vaccine, a knowledge of nAbs, binding affinities, binding kinetics, and B and T-cell responses is key. It is important to characterize antibodies made against SARS-CoV-2 to understand the range of nAbs generated and the targeted immunogenic sites. It is equally important to characterize the T-cell response, as this is crucial in the primary and memory viral immune responses (14, 15).

Some initial work focused on finding antibodies produced against SARS-CoV that could cross-react and ideally neutralize SARS-CoV-2; outcomes have been variable and inconsistent. While one study found that human SARS-CoV mAb S309 could neutralize both SARS-CoV and SARS-CoV-2 pseudoviruses, another study found that even though there was cross-reactivity with SARS-CoV by SARS-CoV-2-specific antibodies from the sera of 23 COVID-19 patients, neutralization of SARS-CoV-2 was only observed with SARS-CoV-2-specific antibodies (16, 17). Further, one study found that the single-domain antibody VHH-72-Fc neutralized both SARS-CoV and SARS-CoV-2 S VSV pseudoviruses, while another found that antibodies B5, B38, H2, and H4, which were raised against SARS-CoV and selected against SARS-CoV-2 in mice, bound to the RBD and led to variable disease outcomes (18, 19).

The ideal intervention is a SARS-CoV-2 vaccine. While many of the vaccine candidates harness only the RBD, it is worthwhile to examine S2 as a potential immunogen due to the precedent of highly effective gp41 neutralization in HIV-1, the structural conservation of S2, the neutralization and cross-reactivity potential of S2 antibodies, and the T-cell response.

## The Case for S2: the Parallels in Structure and Function Between HIV-1 and SARS-CoV-2

HIV-1 can be used as a precedent to highlight the potential importance of SARS-CoV-2 S2 (20). In HIV-1, gp120 is structurally analogous to the S1 subunit that harbors the RBD; both are exposed and involved in binding respective receptors. Similarly, HIV-1 gp41 is structurally analogous to S2; both are less exposed and their structural rearrangements following receptor engagement are central driving forces for fusion between the viral and host cell membranes. While there are a greater number of known nAbs against gp120, those targeting gp41 are also potent (21). One of the struggles in HIV-1 vaccine development is the higher sequence diversity of gp120 than gp41 (22, 23). Similarly, S1 has greater sequence diversity than the more conserved S2 (12, 24). In fact, a comparison of sequence diversity between the SARS-CoV strain Tor2 and the SARS-CoV-2 strain from Wuhan, China found that the RBD was less conserved, having only a 64% identity match, while the fusion domain of S2 was more conserved, having a 90% identity match (9).

From another viewpoint, HIV-1 envelope (Env) has several glycans, and interestingly, a group of Fab-dimerized glycan-reactive HIV-1-induced broadly nAbs (bnAb) could bind to S2 (25). The glycan shield, mainly composed of oligomannose, helps HIV-1 avoid detection by the immune system (26). Notably, SARS-CoV and MERS have fewer glycans and more complex N-glycans and complex, hybrid, and high mannose-glycans than oligomannose glycans as in HIV-1 (25, 27). One research group used molecular dynamics simulations of S to examine glycans. S was separated into 3 regions: the head, (residues 1-1140), the stalk, consisting of the HR2 and transmembrane (TM) regions (residues 1141-1234), and the cytoplasmic tail (residues 1235-1273). Fewer glycans were present on the head compared to the stalk, leading to the conclusion that head regions could be easier for antibodies to reach than stalk regions (28). Because glycans can not only adversely affect the immune system's ability to detect and respond to the virus but also prevent antibodies from accessing important antigenic determinants, stability and accessibility of S2 as an immunogen need further study (28, 29). However, given that bnAbs against HIV-1 gp41 still show promise, it is very possible for S2 to be included as a vaccine immunogen.

The bnAbs 4E10, 10E8, DH511, and LN01 interact with the membrane proximal external region (MPER) of gp41 of HIV-1 (21, 30, 31). S2 of SARS-CoV-2 also harbors an MPER-like sequence (32). The MPER is generally poorly accessible, but accessibility increases transiently when Env binds receptors, gp120 is shed, and gp41 undergoes the structural rearrangements that ultimately facilitate viral entry into the cell. While the mechanistic role of MPER in HIV-1 entry remains an active field of pursuit, so does the potential of MPER as an immunogen (30, 33). In an analysis of LN01, it was discovered that the MPER-TM, if placed in the membrane and stabilized, may be a useful immunogen for HIV-1 (31). In view of the potential of gp41 and its subdomains to elicit nAbs, it is certainly worth examining S2 in the same light for vaccine development.

## The Case for S2: Benefits of the Structural Conservation of S2

S2 is more conserved among coronaviruses than S1. Therefore, it may elicit more cross-reactivity compared to S1, specifically the RBD, which is the least conserved region of S (12, 24). Thus, cross-reactive nAbs against S2 are more likely to preexist in populations due to previous exposures to (HCoVs) (13).

Knowledge of S2 conservation can translate to vaccine design. Vaccines that include the fusion peptide (FP) (residues 795-848) and HR2 (residues 1127-1177) sites in their immunogens may induce production of more broadly active nAbs, combat other betacoronaviruses, elicit a stronger and longer-lasting memory response, and reduce the likelihood of sequence-altering mutations that render the vaccine ineffective (13).

Additionally, because S2 is more structurally conserved, it is less prone to accommodate non-synonymous mutations, as there are multiple S2 regions that are required for fusion and thus infection (11). Examples of S2 sites are the FP and HR2 (13). Jaimes et al. found that, compared to the SARS-CoV Tor2 strain, the FP region of SARS-CoV-2 had a 93% matching identity and the HR2 region had a 100% matching identity (9). Conversely, S1 is more likely to retain mutations that could alter the amino acid sequence because these are less frequently consequential to function. One study (34) used a recombinant chimeric VSV/SARS-CoV-2 reporter virus to study SARS-CoV-2 strains that could lead to resistance against antibodies because of variance in the N-terminal domain and the RBD. Research like this is key, as SARS-CoV-2 variants that are resistant to commonly-elicited nAbs are beginning to appear in humans. Accumulation of such neutralization-evasive changes could lead to broader antibody resistance and complicate convalescent plasma and mAb therapies as well as vaccine development due to incomplete protective immunity. nAbs also differ from patient to patient (34). The lower conservation of S1 compared to S2 highlights why S2 is worth examining as part of a vaccine. The fact that S1 is more likely to harbor mutations that could affect the amino acid sequence may have played a role in the pandemic when a D614G variant became prevalent. Amino acid 614 is a part of the carboxy-terminal region of S1 (35). As G614 became increasingly prevalent, it was determined that pseudoviruses bearing the G614 variation grew to higher titers than the wildtype. Analysis via RT-PCR also suggested that viral loads may be greater in patients infected with the G614 variant, though pathogenic severity may not have been proportionally greater (36). Even more recently, SARS-CoV-2 has spread among minks in Denmark. While many mutations have been discovered, there is focus on the Y453F mutant in the RBD, as a preliminary report shows that this mutation increases the affinity of the RBD for the human ACE2 receptor (37, 38). The mink is now a well-known animal reservoir for SARS-CoV-2, and transmission to humans has been demonstrated (39). While further investigation is needed to determine whether the mutations in this mink outbreak could epidemiologically worsen the COVID-19 pandemic, the principle remains that mutations in the RBD may yield more transmissible variants. Therefore, it is important to develop a vaccine that could remain effective if more infectious variants arise.

## The Case for S2: Neutralization and Cross-Reactive Potential of Antibodies Against S2

While there are many nAbs against the RBD of SARS-CoV and SARS-CoV-2, cross-reactive and nAbs against specific S2 sites do exist. This is true even in patients who have never contracted COVID-19, notably in children when seroconversion of HCoVs is the greatest (40, 41). Therefore, harnessing the prior existence of SARS-CoV-2 nAbs in the human population via a vaccine that elicits a memory immune response could be effective.

One study found that 51.5% of the cohort of 33 convalescent samples were sera-reactive to at least one non-SARS-CoV-2 coronavirus, implying that much of the population may have antibodies against conserved coronavirus regions. Further examination found that there were five conserved regions between SARS-CoV-2 and betacoronaviruses hCoV-HKU1 and hCoV-OC43. These five regions were all within S2. The greatest reactivity was in the HR2 region (13). In a study of a recombinant protein of SARS-CoV residues 268-1255, BALB/c antisera could neutralize the SARS-CoV infection of Vero E6 cells. Epitope mapping determined that these neutralizing mAbs were against residues 1143-1157, which is part of the HR2 region. Based on these results, the HR2 region may be a viable vaccine target against SARS-CoV and potentially SARS-CoV-2 (42). These findings could contribute to not only vaccine development but also therapeutics like an antibody cocktail, which could effectively reduce harm caused by escape mutants (43).

In a different study, sera from both HCoV and COVID-19 patients could bind full S of SARS-CoV-2, but only sera from COVID-19 patients could bind S1 alone. This same study found that 12 of the 95 patients who did not have COVID-19 had IgG antibodies that cross-reacted with conserved parts of SARS-CoV-2, particularly S2 and N. Interestingly, one COVID-19 sample collected 16 days after symptoms cross-reacted with S of SARS-CoV-2 but not S1 of SARS-CoV-2, suggesting memory of HCoVs since epitopes other than SARS-CoV-2 S1 are more conserved. Later, this study found that non-COVID-19 sera could neutralize pseudotypes of the S protein with roughly the same efficacy as COVID-19 sera. Also, the correlation between the titer of nAbs and the titer of antibodies against S was greater in non-COVID-19 sera than COVID-19 sera. Antibodies that are reactive against non-SARS-CoV-2 coronaviruses, such as HCoVs, have potential to target cellular entry of SARS-CoV-2 (40).

Further study of patients who had never been infected with SARS-CoV-2 found that 5 out of the 6 patients who had antibodies reactive against S, as determined by fluorescence-activated cell sorting assay (FACS), could neutralize SARS-CoV-2, but those who did not have antibodies against S could not. Preexisting antibodies against conserved regions of the SARS-CoV-2 S protein, which lie in S2, could hinder SARS-CoV-2 entry into cells. Antibodies against conserved epitopes S901-906, S810-816, S851-856, S1040-1044, and S1205-1212 showed the greatest cross-reactivity (40).

Out of a later cohort of 48 individuals between 1 and 16 years old who had never been infected by SARS-CoV-2, at least 21 had antibodies against S. The presence of these antibodies was greatest at age 6, the age of greatest HCoV seroconversion. Sera from those who were younger and had not been infected with SARS-CoV-2 could neutralize pseudotype viruses bearing SARS-CoV-2 S. Two of these sera could also neutralize genuine SARS-CoV-2 (40). The COVID-19 pandemic has been notable for producing less severe symptoms in children (44). Perhaps this is due to greater seroconversion of HCoVs in childhood, allowing for a stronger immunological memory and thus a greater number of nAbs against epitopes conserved across coronaviruses.

A different study found that 86% of those without SARS-CoV-2 had antibodies against S2 of SARS-CoV-2. Memory B-cells (MBC) against S2 were not found, leading to the hypothesis that the threshold of S2 MBC detection was too high given the levels. This hypothesis was consistent with the finding that most of the convalescent sera had higher levels of anti-S2 antibodies than anti-RBD antibodies, which could have been caused by provocation of S2 MBCs. It was hypothesized that MBCs could produce IgG protective against future infection of not only SARS-CoV-2 but also other coronaviruses (41).

Utilizing S2 could improve the effectiveness of a COVID-19 therapy or vaccine, as demonstrated by the prevalence of cross-reactive and nAbs in non-COVID-19 sera and children having both the highest seroconversion of HCoVs and interestingly low rates of COVID-19 symptoms. The fact that conserved epitopes in S2 have already been found makes an effective vaccine that includes an S2 epitope even more tenable.

## The Case for S2: the Importance of Conserved Epitopes in the T-Cell Response

Analyzing not only the antibody responses but also the T-cell responses is crucial to understanding the immune response to SARS-CoV-2. Interestingly, a study of 5 different cohorts from Germany, Singapore, Netherlands, UK, and the USA found that 20 to 50% of individuals who never had SARS-CoV-2 had reactive T-cells against sequences of SARS-CoV-2 (45–49). It was thought that this could be due to prior exposure to common cold coronaviruses. It is possible that SARS-CoV-2 infection in someone with preexisting T-cell memory may lead to different symptoms than in someone without T-cell memory (20). This could affect how not only an individual but also an entire population would react to a SARS-CoV-2 vaccine. Mateus et al. found that 10 out of 42 memory CD4+ T cell lines from all four common cold coronaviruses could cross-react with conserved regions of SARS-CoV-2, particularly at the S, N, nsp13 helicase, nsp12 RNA dependent RNA polymerase, and nsp8 peptide cofactor regions (20, 50). Further inspection of sequence homology and correlation to cross-reactivity of T-cells at these sites showed that 33–40% of homology led to cross-reactivity in 1% of cases, 47–60% homology led to cross-reactivity in 21% of cases, and greater than 67% homology led to cross-reactivity in 57% of cases (20). The levels of T-cells against the common cold coronaviruses in populations could be even more important than levels of circulating antibodies because antibodies to the common cold coronaviruses tend to significantly decrease over a few months post-infection (51). If greater sequence homology led to greater cross-reactivity at sites of non-structural proteins, it is possible that it could lead to cross-reactivity at structural protein sites, such as S2. More analysis needs to be completed to determine whether cross-reactive T-cells reduce infectivity of SARS-CoV-2, as results have varied across different studies (49, 52). However, this is not out of the question, as T-cell responses to SARS-CoV structural proteins studied in mice significantly reduced viral burden (53–55).

While the B-cell response and antibody production are extremely important, the T-cell response, particularly T-cell memory, is also crucial for a strong and long-lasting response to an antigen. If existing T-cell memory due to common cold coronaviruses can combat SARS-CoV-2, a vaccine that elicits that memory could prove to be very effective.

## Discussion

Currently, there appears to be a greater number of characterized nAbs against the RBD than S2. Even if this is true and even if the RBD is considered to be a more exposed, first-line target, S2 may still be beneficial as a vaccine immunogen from an individual and epidemiological standpoint, especially if it is less prone to retain mutations and will elicit a memory immune response in much of the population. Since many potent bnAbs against gp41 of HIV-1 have been discovered, further research on the structurally-similar S2 as a potential immunogen is warranted. S2 is less likely than S1 to harbor mutations, and thus targeting S2 in a therapeutic or vaccine may reduce the risk of vaccine-evasive SARS-CoV-2 variants, which could improve epidemiological outcomes. Furthermore, due to conservation of S2 and prior population exposure to common cold coronaviruses, nAbs and memory B- and T-cells against SARS-CoV-2 may already exist in individuals who have never been infected by SARS-CoV-2. In contrast to the above benefits, there are possible limitations. One potential limitation is the glycosylation pattern of S2, but SARS-CoV-2 is less glycosylated than HIV-1, and potent bnAbs against HIV-1 still exist. Another is possible antibody-dependent enhancement, but this is speculative at present (56). A third one is antigen competition, but this is also speculative at present. Lastly, some S2 epitopes are only transiently exposed during the fusion process. Nonetheless, discovery of bnAbs against gp41, which is also transiently exposed during fusion, the benefits of the more-conserved S2, and previous exposure to HCoVs all argue in favor of further exploration of a vaccine that includes S2 as an immunogen. Experimentally, existing assays can and have been used to characterize T-cell responses and antibody reactivity, affinity, and kinetics. These assays include ELISPOT, ELISA, neutralization assays, and the less commonly used, yet advantageous, surface plasmon resonance (SPR) (57–59). A similar approach was successfully used in SARS-CoV-2 research to characterize immunized rabbit sera (58). Existing methods can and should assess the utility of a vaccine that includes S2 as an immunogen. A strong case exists for the scientific community to explore and harness the potential benefits of S2.

## Data Availability Statement

Publicly available datasets were analyzed in this study.

## Author Contributions

PS, a medical student, performed the background research, formulated the perspective, and wrote all the manuscript drafts. GAC, EPC, and IC helped shape the idea, provided suggestions and guidance, and suggested edits for the drafts. GAC made the figure and wrote the caption. All authors have contributed to this article and have approved it for submission.

## Conflict of Interest

The authors declare that the research was conducted in the absence of any commercial or financial relationships that could be construed as a potential conflict of interest.
